# Weight misperception amongst youth of a developing country: Pakistan -a cross-sectional study

**DOI:** 10.1186/1471-2458-13-707

**Published:** 2013-08-02

**Authors:** Muhammad Danish Saleem, Gulrayz Ahmed, Juwaria Mulla, Syed Sami Haider, Mustafa Abbas

**Affiliations:** 1 Medical Student, Dow Medical College, Dow University of Health Sciences, Baba-E-Urdu Road, Karachi, Pakistan

**Keywords:** Weight misperception, BMI, Pakistan, Undergraduate students, Youth, Overweight, Underweight, Overestimation, Underestimation

## Abstract

**Background:**

*Weight misperception* is the discordance between an individual’s actual weight status and the perception of his/her weight. It is a common problem in the youth population as enumerated by many international studies. However data from Pakistan in this area is deficient.

**Methods:**

A multi-center cross-sectional survey was carried out in undergraduate university students of Karachi between the ages of 15–24. Participants were questioned regarding their perception of being thin, normal or fat and it was compared with their Body Mass Index (BMI). Measurements of height and weight were taken for this purpose and BMI was categorized using Asian cut offs. Weight misperception was identified when the self-perceived weight (average, fat, thin) did not match the calculated BMI distribution. Chi square tests and logistic regression tests were applied to show associations of misperception and types of misperception (overestimation, underestimation) with independent variables like age, gender, type of university and faculties. P-value of <0.05 was taken as statistically significant.

**Results:**

42.4% of the total participants i.e. 43.3% males and 41% females misperceived their weight. Amongst those who misperceived 38.2% had overestimated and 61.8% had underestimated their weight. Greatest misperception of was observed in the overweight category (91%), specifically amongst overweight males (95%). Females of the underweight category overestimated their weight and males of the overweight category underestimated their weight. Amongst the total participants, females overestimated 8 times more than males (OR 8.054, 95% CI 5.34-12.13). Misperception increased with the age of the participants (OR 1.114, 95% CI 1.041-1.191). Odds of misperception were greater in students of private sector universities as compared to public (OR 1.861, 95% CI: 1.29-2.67). Odds of misperception were less in students of medical sciences (OR 0.693, 95% CI 0.491-0.977), engineering (OR 0.586, 95% CI 0.364-0.941) and business administration (OR 0.439, 95% CI 0.290-0.662) as compared to general faculty universities.

**Conclusion:**

There was marked discrepancy between the calculated BMI and the self-perceived weight in the youth of Karachi. Better awareness campaigns need to be implemented to reverse these trends.

## Background

*Weight misperception* is the discordance between an individual’s actual weight status and the perception of his/her weight [1]. Weight misperception may be broadly divided into under-estimating or over-estimating ones weight. Underestimation or underweight misperception is considering oneself of a lower weight than they actually are, as determined by Body Mass Index (BMI) [2]. Overestimation or overweight misperception is considering oneself of being a higher weight than they actually are, as determined by BMI [2].

The youth (age group of 15–24), according to the 2007 United Nations Development Program statistics form a substantial 21.8% (36 million) of the total population of Pakistan [3]. In spite of this high percentage, very few epidemiological studies have been conducted on this age group in our country.

Obesity is emerging as a public health problem. Traditionally thought of as a disease of adulthood, it is finding its way into adolescents and the youth population. Rehman et al. reported an overwhelming 18% overweight distribution amongst tenth grade adolescents of Karachi [4]. Obesity or being overweight increases the risk of adverse cardiovascular outcomes, type 2 diabetes mellitus, cancers and even death [5]. On the other end of the spectrum, being underweight poses serious problems, especially in a developing country like Pakistan. A report on the nutritional status of Pakistan states a high prevalence of the population as underweight, majority of which are women of reproductive ages [6]. Being underweight affects both sexes alike, however the effects are more harmful in the female youth and young mothers with implications such as anemia, infertility or complications in pregnancy endangering both the life of the mother and of their babies [7].

Research studies have demonstrated that self-awareness of being overweight/ obese is an essential factor in attempting to lose weight [8]. Consequently, weight loss is associated with decreased health risk and can substantially reduce the incidence of hypertension and cardiovascular diseases [9]. Intake of proper food and diet with regular eating hours and awareness in the general population about the harms of being underweight and undernourished can help improve the complications associated with undernourishment [7].

The key to health and weight management is proper weight perception. Numerous studies show that overweight and underweight misperception is a dangerous albeit common problem in the youth around the globe. Data from the 2007 Minnesota student survey of 87,418 high school students showed that a quarter of their students had improper weight perception which was more prevalent in males, with girls more likely to overestimate and boys more likely to underestimate [2]. The CDC’s National youth risk behavior survey of 2007 consisting of 12,853, 9^th^ to 12^th^ grade students in U.S public and private schools showed that 23% of overweight girls and 40% of overweight boys had misperceived [10]. A Turkish study in high school students aged 14 to 21 years from Adana, a southern city of Turkey, during 1999 also reported high weight misperception [11]. This study on 2,352 students incorporated the Turkish version of youth risk behavior survey questionnaire and reported that girls misperceived themselves to be overweight and obese as compared to boys and displayed a greater percentage of weight loosing behaviors [11].

The youth, at the brink of joining professional life are a country’s major asset and any health problems identified and resolved at this stage would benefit the adults of future years. Weight misperception has previously been identified in the adults of Pakistan. A survey conducted on 493 adults in Karachi, Pakistan demonstrated a poor agreement between self-perception and actual BMI with a 73% and 50% misperception amongst the obese and overweight participants respectively [12]. However no comprehensive survey demonstrating weight perception in the youth of Pakistan has been conducted.

For our sample we targeted the youth population of Karachi studying in various undergraduate universities. The purpose of our study was to obtain weight misperceptions amongst the youth population by comparing their actual weight categories based upon calculated BMI with their self-perception and to explore some relationships of this misperception with gender, type of university (public or private) and faculties (general, engineering, medical sciences and business administration).

## Methods

### Study design

We conducted a multi-center cross-sectional study in different undergraduate universities of Karachi, Pakistan, during the months of January and February 2012. Sixteen hundred individuals from six universities were interviewed. Universities were selected on the basis of systematic sampling from the list of 33 public and private sector universities of Karachi, recognized by the Higher Education Commission of Pakistan. Universities were arranged in alphabetical order. The first university was selected randomly, while the remainder five universities were chosen with a gap of five universities each. For the purpose of data collection, the sample was distributed amongst the individual universities by proportion of their estimated student population. Simple random sampling using the student roll numbers was used to select the study participants.

### Sample population

Both private and government undergraduate universities were selected to ensure participation of students from all socioeconomic backgrounds and localities of Karachi, producing a better representation of the youth population. Even though these universities do cater to a meager amount of international students, our study involved data collection only from Pakistani nationals. Karachi is a mega-city and the economic capital of the country. It hails inhabitants from all cultures and migrants from all cities of the country. Thus participants selected form an ideal representation of all the students of Pakistan. All participants, male and female, present in the university during the data collection period that fell in the age range of 15 - 24 [3] years were included. Any student above and below these ages were excluded. We approached a total of 1,600 students, out of which 1,400 agreed to participate in the survey (response rate 87.5%).

### Data collection tool

We designed a short structured, close-ended questionnaire in English, which is the medium of instruction in colleges and universities of Pakistan. It is comprised of the variables and demographics. The students were approached by the investigators, the procedure of the study was explained to them and informed signed consent was obtained. The questionnaire was administered in the form of an interview. The interview was followed by the height and weight measurements of the participants. Participants were interviewed prior to the procedure of physical measurements to reduce bias in reporting misperception. A meeting of the investigators was held prior to the data collection procedure to maintain uniformity in its administration.

### Independent variables

The weight (in kilograms) and height (in centimeters) of the participants was measured by the investigators. BMI was calculated from height and weight measurements and then categorized into underweight (<18.5), normal (18.5-22.9), overweight (23–24.9) and obese (≥ 25.0) based on the new Asian cutoffs [13].

Self-perception of body weight was determined by the question: Do you consider yourself as a) average b) thin c) fat. This question was structured in plain English so that all the participants could answer correctly.

Participant demographics namely age, gender, faculty (general, engineering, medical sciences and business administration) and type of university (public or private) were considered as the other independent variables. Ethnicity was not reported as all the participants belonged to the South Asian ethnicity.

### Dependent variables

The responses were then compared to the calculated BMI to determine misperception. For underweight participants, misperception was considered when the response to the question was average or fat. For normal participants, misperception was considered when the response was thin or fat. For overweight/obese participants, misperception was considered when the response was average or thin. The types of misperception (overestimation or underestimation) were also determined.

### Statistical analysis

All data were entered and analyzed on SPSS v.18. Mean and standard deviation was calculated for all the continuous variables like age and BMI. Frequencies and percentages were reported for all the categorical variables such as gender, BMI distribution, type of university and faculties. T-test was used to measure differences in means for continuous variables between males and females. Chi square test was applied to determine association for the categorical variables with gender, misperception and types of misperception. Two logistic regression models were applied to see the effect of demographic variables for both misperception and types of misperception (overestimation vs underestimation). Gender was not found to be statistically significant with misperception in the regression model. Meanwhile gender was the only variable found to be statically significant with types of misperception in the regression model. P-value of less than 0.05 was taken as statistically significant.

### Ethical considerations

Data from female participants was collected by the female investigators keeping in view the social and cultural setup of our country. The questionnaires were kept anonymous to ensure confidentiality and were numbered serially. Informed signed consent was obtained from all the participants and the study protocol was reviewed and approved under the Dow University Ethical Review Board.

## Results

### Demographics

All participants were single, Pakistani nationals from the ages 15–24 years and enrolled in a university. The mean age of the participants was 19.59 (±1.7) years. Eight hundred and fifty six (61.1 %) of the participants were male and 544 (38.9%) were female. The mean BMI of the participants was 20.73 (±3.5), with 390 (27.9%) under-weight, 715(51.1%) normal, 154 (11%) overweight and 141 (10.1%) obese. The t-test for the difference of means between the two genders for both age and BMI was significant (p-value<0.001). Table 1 shows the gender based distribution of the independent variables.

**Table 1:** Gender based distribution of independent variables

**Independent variables**		**Male**		**Female**		**p-value (chi-square)**
		**N = 856**		**N = 544**		
		**N**	**%**	**N**	**%**	
**1. University type**	Public sector	486	56.8	289	53.1	0.186
	Private	370	43.2	255	46.9	
**2. Faculties**	General (Art+ Science)	304	35.5	174	32	<0.001
	Engineering	227	32.4	123	22.6	
	Medical sciences	148	17.3	199	36.6	
	Business and Administration	127	14.8	48	8.8	
**3. BMI**	Underweight	159	18.6	231	42.5	<0.001
	Normal	478	55.8	237	43.6	
	Overweight	109	12.7	45	8.3	
	Obese	110	12.9	31	5.7	
**4. Self-perception**	Thin	245	28.6	113	20.8	0.001
	Average	537	62.7	396	72.8	
	Fat	74	8.6	35	6.4	

Table shows percentage and frequency distribution of different independent variables as observed in the two genders and the respective p-values of their chi square tests with gender.

### Self-perception

Three hundred and eighty five (25.6%) participants considered themselves thin, 933 (66.6%) considered themselves average and 109 (7.8%) considered themselves fat. Most females (72.8%) self-perceived themselves to be average. Compared to females, more males considered themselves to be either thin or fat. The association between self-perception and gender was found to be significant (p-value = 0.001). (Refer to Table 1).

### Weight misperception

Misperception was identified when the self-perceived weight (average, fat, thin) did not match the calculated BMI distribution (underweight, normal or overweight/obese). There was poor agreement between self-perception and calculated BMI, and 594 (42.4%) of the total participants misperceived their weight. Greatest misperception was observed amongst the participants of the overweight category (91%) and the least misperception was observed in the participants of the normal weight category (24.2%). Three hundred and seventy one (43.3%) of the total males and 223 (41%) of the total females misperceived their weights. The association between misperception and gender was not found to be significant (p-value = 0.406). Table 2 shows association of weight misperception with independent variables.

**Table 2:** Association of misperception with independent variables

**Independent variables**		**Yes**		**No**		**p-value (chi-square)**
		**N = 856**		**N = 544**		
		**N**	**%**	**N**	**%**	
**1. Gender**	Male	371	43.3	485	56.7	0.406
	Female	223	41	321	59	
**2. BMI**	Underweight	214	54.9	176	45.1	<0.001
	Normal	173	24.2	542	75.8	
	Overweight	140	90.9	14	9.1	
	Obese	67	47.5	74	52.5	
**3. University type**	Public Sector	298	38.5	477	61.5	0.001
	Private	296	47.4	329	52.6	
**4. Faculty**	General (Art+ Science)	200	41.8	278	58.2	0.001
	Engineering	192	48	208	52	
	Medical sciences	149	42.9	198	57.1	
	Business Administration	53	30.3	122	69.7	

Table shows distribution of the independent variables with weight misperception (yes or no). P-values of chi square tests of misperception with the independent variables have been quoted as well.

**Figure 1:**
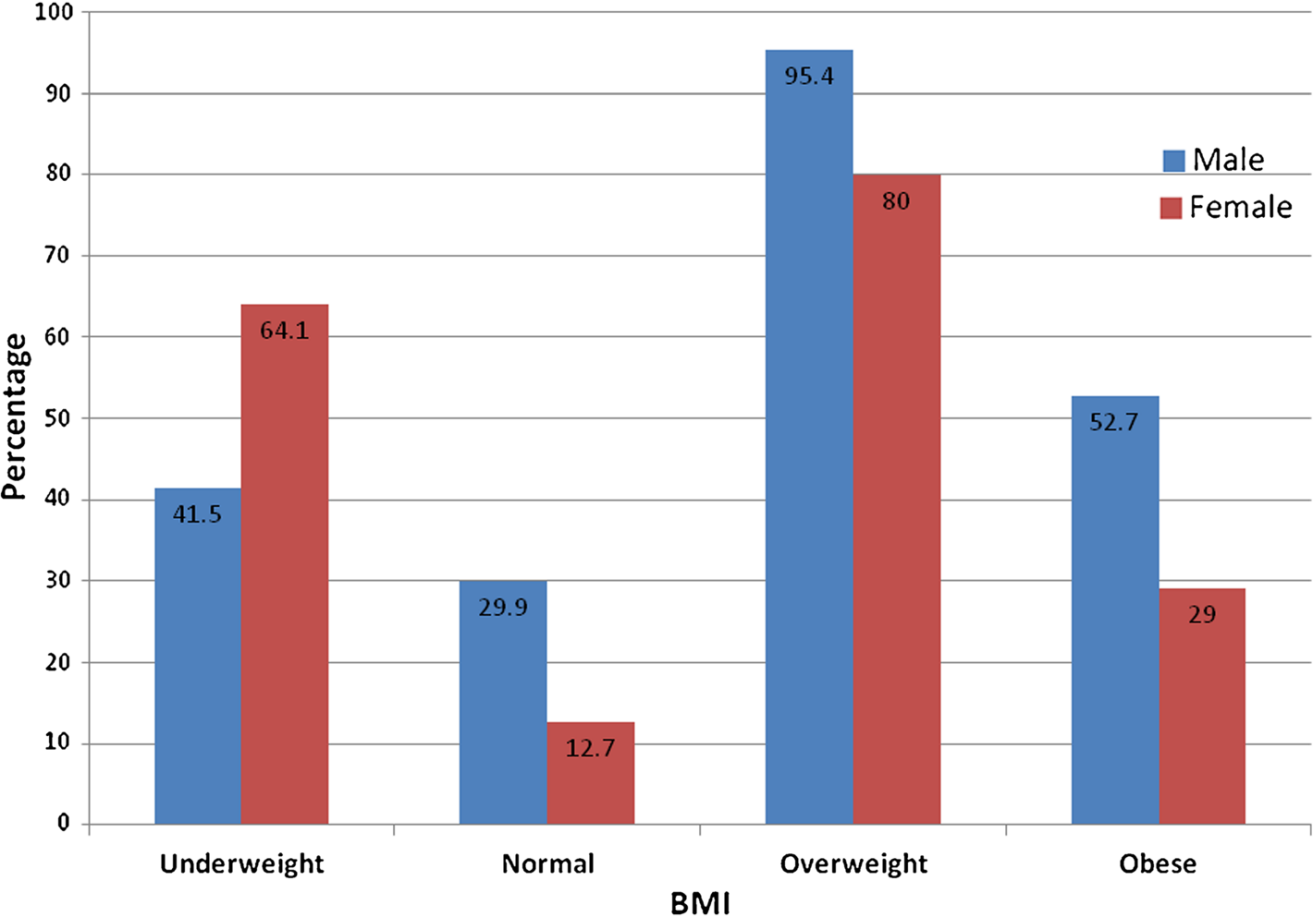
**Distribution of weight misperception in the participants according to BMI and Gender.** Figure shows the distribution of misperception of weight in the participants. The total participants have been categorized into the four categorizes of BMI which are underweight, normal, overweight and obese. Each of these categories is further divided into male (blue) and female (red) on the basis of gender. This figure shows the percentage of misperception reported, calculated from the total (N) males and females who participated in the study.

Figure 1 shows the distribution of misperception between the two genders according to BMI of the participants. The highest percentage of weight misperception was observed in the overweight males (95.4%) and the lowest percentage was observed in normal weight females (12.7%).

Logistic regression for weight misperception showed that misperception increased with the increasing age of participants (OR 1.114, 95% CI 1.041-1.191). The chances of misperception were greater in students of private sector universities as compared to public sector (OR 1.861, 95% CI 1.29-2.67). The odds of misperception were also higher in students of the general faculty as compared to engineering, medical sciences and business administration faculties. (Refer to Table 3).

**Table 3:** Associations between misperception and age, type of university and faculty (results from logistic regression)

**Independent variables**		**Odds ratio**	**95% CI**		**p–value (chi-square)**
**1. Age**		1.114	1.041	1.191	0.002
**2. Type of university**	Public sector	1	-	-	-
	Private university	1.861	1.294	2.677	0.001
**3. Faculty**	General(Arts+ Science)	1	-	-	-
	Engineering	0.586	0.364	0.941	0.027
	Medical sciences	0.693	0.491	0.977	0.036
	Business administration	0.439	0.290	0.662	<0.001

Table shows the associations for logistic regression analysis of misperception with the independent variables. Only those variables with statistically significant p-values are shown. *CI* Confidence interval.

### Types of misperception

Misperception was classified as either overestimation or underestimation. Amongst those who misperceived, 227 (38.2%) had overestimated their weights and 367 (61.8%) had underestimated their weight status. Majority of the total overestimation was observed amongst the participants of the underweight category (n = 214, 94.3%), while majority of the total underestimation was observed from the participants of the normal (43.6%) and overweight category (38.1%). The association between the types of misperception and gender was found to be significant (p-value < 0.001). Underestimation was more commonly observed among males (296 of the 371 misperceiving males). In contrast overestimation was more commonly observed among females (152 of the 223 misperceiving females). Logistic regression for types of misperception showed that males had an eight times more chance of underestimation as compared to females (OR 8.054, 95% CI 5.34-12.13).

Table 4 shows the distribution of overestimation and underestimation in the participants. More underweight females overestimated their weight as compared to underweight males (27.2% vs 7.7%). Greater percentage of normal weight males underestimated their weight as compared to the normal weight females (15.6% vs 4.8%). Similarly more overweight and obese males underestimated their weight as compared to overweight (12.1% vs 6.6%) and obese (6.8% vs 1.6%) females.

**Table 4:** Distribution of overestimation and underestimation of weight according to BMI and Gender

**BMI**	**Overestimation**				**Underestimation**			
	**N = 227**				**N = 367**			
	**Male**	**%**	**Female**	**%**	**Male**	**%**	**Female**	**%**
**Underweight**	66	29	148	65.2	-	-	-	-
**Normal**	9	4	4	1.8	134	36.5	26	7.1
**Overweight**	-	-	-	-	104	28.3	36	9.8
**Obese**	-	-	-	-	58	15.8	9	2.5

Table shows the distribution of overestimation and underestimation of weight in the participants on the basis of BMI and gender. Overestimation and underestimation is further divided into subcategories formed by BMI and gender. Percentages have been calculated from total number (N) for either overestimation or underestimation. Highest values have been highlighted.

## Discussion

Weight misperception was a highly prevalent finding in our study with as much as 42.4% overall misperception seen in the total youth population. We witnessed a considerable fraction of individuals in the overweight and obese categories erroneously reporting their weight status; hence showing a high level of misperception among overweight and obese participants. This is of concern as literature has shown that correct perception of one’s weight is strongly associated with positive efforts to lose weight and maintain a healthy life style. Skinner et al. [14] and Jones et al. [15] reported that those who misperceived their weight had less weight concerns, less control over distress and emotional overeating, more often indulged in improper dietary intake (including fast food and soda beverages) along with inappropriate snack timings and had more sedentary and stationary lifestyles. Edwards et al. reported that overweight youngsters who precisely perceived their weight were more likely to lose weight and indulge in exercises, all the while consciously consuming fewer calories [10].

Our results regarding weight misperception are similar to those seen in studies from both developed and developing countries. High proportions of misperception regarding weight in the youth populations were reported from USA [2], Turkey [11], China [16], Mexico [17], Spain [18] and Malaysia [19] where high percentages of underweight individuals misperceiving themselves as normal was observed.

Our study showed that misperception increased with the increasing age of participants (OR 1.114, 95% CI 1.041-1.191). Similar result has also been shown by a study conducted in Spanish adolescents which reported a higher mean age for those who misperceived as compared to the others [18]. Chinese [16] and Mexican [17] studies have also reported underestimation to be associated with older age.

The chances of misperception were greater in students of private sector universities as compared to public sector. It was also noted that students of the general faculty misperceived more as compared to engineering, medical sciences and business administration faculties. Students from the public sector science faculties had the least misperception of weight as compared to the other undergraduate students in our sample.

The two most important findings in our study were the high prevalence of misperception amongst underweight females as being normal or overweight (overestimation) and the misperception amongst overweight and obese males as being normal or underweight (underestimation).

### Overestimation of weight in females

Overestimation of weight was a less common finding in our setting; nevertheless one group outnumbered the rest i.e. underweight females overestimating their weights. It would not be a surprise if this misperception was accompanied by unhealthy weight reduction practices. Talamayan et al. reported that a significant portion of normal weight adolescents misperceive themselves as overweight and engage in unhealthy weight control behaviors. Females (16.8%) outnumbered males (6.8%) in practicing at least one such unhealthy behavior (use of diet pills, laxatives, and fasting) in the past 30 days [20]. Another study conducted in students of Adana reported 35.5% participants as wanting to lose weight, with weight losing intentions and interventions (diet, provocative vomiting and 24-hours starving) more common amongst girls [11]. Indeed anorexia has been noted as an important problem in female students of Pakistan [21], even though a relationship between anorexia and weight misperception still needs to be determined in Pakistani students.

Females tend to be more sensitive towards their weight status and are likely to take social, peer and family pressures more seriously than their male counter-parts. A study conducted on female college students showed that many females identify media as a source of motivation and pressure, encouraging them to be of certain weight [22]. A study conducted amongst university students of Karachi had similar results with higher percentages of underweight women perceiving themselves as normal [23]. This study reported that vast majorities of females were influenced by images of thin models and consequently strived to achieve this ideal body image even when they were majorly underweight [23]. Wardle et al. showed that about half of the female students from 22 different countries considered themselves overweight and were trying to lose weight despite the fact that a majority of them had normal BMI values [24].

### Underestimation of weight in males

In our study majority of overweight and obese males considered themselves as having a normal or average weight. The preference of males for a heavier body structure while perceiving themselves to be average when most were overweight can have many reasons. The abundance of ‘muscular’ models portrayed in the media [25] coupled with a relative absence of particularly thin male models could be one of the causes. A study amongst youth of the United States showed that males tend to perceive themselves as normal whilst they were actually overweight [2]. Similar studies in residents of Guangdong province of China showed that males were more likely to underestimate weight than females (25.8% vs. 8.5%) [15]. Customarily, increased body weight has been an emblem of health and wealth in a variety of populations [26]. This is especially true for the Indian subcontinent where people still consider weight to be associated with wealth, prosperity and good health. This may lead them to misperceive their weight as normal which can put them at a higher risk for multiple diseases [27].

Bhanji et al. reported that few factors associated with underestimation of weight were male gender, being happy with ones’ weight and not knowing one’s ideal weight [12]. Indeed inadequate knowledge of ideal body weight is also a major contributing factor in weight perception, especially in our social setup, where school heath and health education is not stressed upon. A study from New Delhi, India reported nearly half of the overweight or obese students misclassifying their weight status [28], statistics very similar to our own for the same socioeconomic and cultural background.

### Solutions

Observation of misperception at the ends of the spectrum is rather alarming due to adverse psychological and health related outcomes as severe as suicide, reported by Chinese [29] and Korean studies [30]. Therefore it is important for health professionals to identify both underweight and overweight/obese, educate them about the health risks of their weight and advise them appropriate strategies for gaining or losing weight. The weight loss or gain regime should be closely monitored as drastic weight loss or gain might have adverse effects. More importantly, the interventions made to correct the weight should be carefully designed to protect against body image disorders, eating disorders and emotional distress. Moreover wide scale campaigns need to be undertaken to prevent unhealthy weight trends observed by initiating healthy eating habits and advocating adequate exercise in children [31], adolescents [10], the youth [8] and adults [5]. School health should also be improved with emphasis on educating the students about ideal weight and healthy weight maintenance.

### Strengths and limitations

There exists no data on such an age group in our country. Pakistan being the 6th most populous nation of the world, can give a good perspective of the developing world. The most significant achievement of this study was the objective calculation of BMI of the participants.

There were some limitations of our study as well. The sample cannot be a true reflection of the entire youth population of Pakistan, especially the rural population where many do not have access to university education. The terminology ‘fat’ which was part of the question assessing self-perception may be a limiting factor in our study. ‘Fat’ is a subjective word and may be difficult for people to accept. A major limitation with using BMI scale in measuring up male body was that it does not consider increased muscle mass which could have been a contributing factor of raised BMI in some males. Moreover it could be possible that that these muscular males who ‘misperceived’ themselves as normal weight would in fact not be misperceiving, rather the BMI scale was wrongly classifying them as overweight.

## Conclusion

There was marked discrepancy between the calculated BMI and the self-perceived weight in the youth of Karachi. There was overestimation of weight by underweight females and underestimation by overweight males. Better awareness campaigns need to be implemented to reverse these trends.

## Abbreviations

BMI: Body mass index.

## Competing interests

The authors declared that they have no competing interests.

## Authors’ contributions

MDS contributed in conceptualization of study, data collection, data analysis, initial and final draft writing.GA contributed in conceptualization of study, data collection, data analysis and final draft writing. JM contributed in literature review, data collection and final draft writing. SSH and MA contributed in initial draft formation, data collection, data verification, data entry and review and editing of the final draft. All authors read and approved the final manuscript.

## Pre-publication history

The pre-publication history for this paper can be accessed here:

http://www.biomedcentral.com/1471-2458/13/707/prepub
